# Moderation in All Things

**Published:** 1881-04

**Authors:** 


### MODERATION IN ALL THINGS.

NO lesson is of greater importance than that which teaches moderation in all things. The rule applies with equal force to mind and body. Over-zeal in our efforts to influence others, and bring them over to our way of thinking, is apt to defeat its object by rendering people suspicious of the motive, while quiet argument wins on its merits, and, if there is a selfish motive, conceals it. Many persons can, perhaps, remember having had the whole current of their lives changed by listening to a calm suggestion bearing the impress of disinterestedness, while no sensible man ever had his political faith warped by the screams of a stump orator. A Frenchman can give expression to the bitterest sarcasm, the deepest hatred and the greatest contempt by a mere shrug of the shoulders. Extremes should be avoided. A calm, frank, truthful expression of one's views is always best. So in regard to physical laws and the laws of health—extremes are to be avoided. Every imaginable condition illustrates this truth. Opposite conditions often produce like results. To illustrate, severe labor is the opposite of indolence, yet both are destructive to health. Regular and sufficient occupation is the mean between these extremes, and is favorable to long life. Certain diseases result also from opposite causes, the most common of which is heart disease.

Persons of sedentary habits, who eat heartily of rich food, make so much blood that the elimination of waste material from the system is not rapid enough to maintain the equilibrium; consequently there is an excess of blood to be circulated, which over-works the heart; this strain, long continued, results in disease. Precisely the same thing may result from an opposite course. If the nourishment is insufficient, the circulation becomes sluggish, the action of the heart feeble, and its walls dilated and diseased. And so with the brain. There are quite as many cases of apoplexy from over abstemiousness as from the opposite cause. Thus also with cogestions and inflammations affecting the various vital organs; the rule of like results from opposite causes holds good. These facts should teach us to avoid extremes, not only in our methods of living, but in matters of opinion also.

---

Some years ago the French ministry addressed a circular to all the prefects, desiring them to institute inquiries as to the conditions which appeared peculiarly to favor longevity in their several districts, and the replies are said to have almost unanimously indicated as the leading elements or influences great sobriety, regular labor and usually in the open air, daily exercise short of fatigue, early hours, a comparatively well-to-do life, calmness of mind in meeting troubles, moderate intellectual powers and a family life. The beneficial influence of marriage on the duration of life is universally admitted, and remarriage does not seem to be unfavorable. The prefects also indicate heredity as a frequent cause, and the influence of climate is likewise admitted; this latter, however, is separable with difficulty from other causes which may be operating simultaneously; but if all things were otherwise equal, it would seem that southern are less favorable to longevity than northern climates.

---

A minister at Ayr, Scotland, recently introduced the following petition into his Sunday prayer: "O Lord, bless the Established Church, and the Free Church, and the United Presbyterian Church, and all the other churches. Thou knowest the various nicknames, Lord, by which they are called; bless them all."

